# AGIA Tag System for Ultrastructural Protein Localization Analysis in Blood-Stage *Plasmodium falciparum*

**DOI:** 10.3389/fcimb.2021.777291

**Published:** 2021-12-15

**Authors:** Masayuki Morita, Bernard N. Kanoi, Naoaki Shinzawa, Rie Kubota, Hiroyuki Takeda, Tatsuya Sawasaki, Takafumi Tsuboi, Eizo Takashima

**Affiliations:** ^1^ Division of Malaria Research, Proteo-Science Center, Ehime University, Matsuyama, Japan; ^2^ Department of Parasitology and Tropical Medicine, Graduate School of Medical and Dental Sciences, Tokyo Medical and Dental University, Tokyo, Japan; ^3^ Division of Proteo-Drug-Discovery, Proteo-Science Center, Ehime University, Matsuyama, Japan; ^4^ Division of Cell-Free Sciences, Proteo-Science Center, Ehime University, Matsuyama, Japan

**Keywords:** malaria, *Plasmodium falciparum*, AGIA tag system, organelle, immunoelectron microscopy

## Abstract

Precise subcellular localization of proteins is the key to elucidating the physiological role of these molecules in malaria parasite development, understanding of pathogenesis, and protective immunity. In *Plasmodium falciparum*, however, detection of proteins in the blood-stage parasites is greatly hampered by the lack of versatile protein tags which can intrinsically label such molecules. Thus, in this study, to develop a novel system that can be used to evaluate subcellular localization of known and novel proteins, we assessed the application of AGIA tag, consisting of 9 amino acids (EEAAGIARP), in *P. falciparum* blood-stage parasites. Specifically, AGIA-tagged ring-infected erythrocyte surface antigen (RESA-AGIA) was episomally expressed in *P. falciparum* 3D7 strain. The RESA-AGIA protein was detected by Western blotting and immunofluorescence assay (IFA) using recombinant rabbit anti-AGIA tag monoclonal antibody (mAb) with a high signal/noise ratio. Similarly, AGIA-tagged multidrug resistance protein 1 (MDR1-AGIA), as an example of polyptic transmembrane protein, was endogenously expressed and detected by Western blotting and IFA with anti-AGIA tag mAb. Immunoelectron microscopy of the RESA-AGIA transfected merozoites revealed that mouse anti-RESA and the rabbit anti-AGIA mAb signals could definitively co-localize to the dense granules. Put together, this study demonstrates AGIA tag/anti-AGIA rabbit mAb system as a potentially useful tool for elucidating the subcellular localization of new and understudied proteins in blood-stage malaria parasites at the nanometer-level resolution.

## Introduction

*Plasmodium falciparum* is the most virulent parasite among five *Plasmodium* species that cause human malaria. Merozoites, one of the parasite invasive forms, deploys a variety of proteins to support its cyclic invasion and egress from human erythrocytes (Cowman et al., 2017; Matz et al., 2020). Thus, to understand the biology, pathogenesis, and protective immunity, and also develop effective intervention tools, in-depth characterization of the key parasite proteins that malaria parasites deploy to invade and develop within the host cells is needed.

Merozoite apical organelles such as rhoptries, micronemes, and dense granules are of major interest in understanding parasite development since they have important roles in merozoite invasion of erythrocytes (Cowman et al., 2017). However, the diminutive nature of the rhoptries (with a diameter of 330–570 nm), micronemes (40–100 nm), and dense granules (120–140 nm) (Torii et al., 1989) in merozoite (1 µm) makes them difficult to study with conventional confocal laser scanning microscopy which has >200 nm resolution. Immunoelectron microscopy (IEM) with a resolution of <1 nm is therefore required to investigate the subcellular localization of the parasite molecules in merozoites. However, in addition to challenges related to generation of quality antibodies against malaria parasite antigens, the harsh sample processing conditions for IEM such as fixation, dehydration and embedding steps may disrupt target epitopes, and consequently loss of reactivity to polyclonal antigen-specific and/or monoclonal antibodies used for probing. To overcome these limitations, transgenic parasites expressing target proteins fused with protein tags such as hemagglutinin (HA), c-Myc, V5, or green fluorescent protein (GFP) have been used in several studies (Downie et al., 2010; Dastidar et al., 2012; Tokunaga et al., 2019; Raj et al., 2020).

However, one study revealed that the GFP tag could influence STEVOR localization (Wichers et al., 2019) but not K13 which was affected by 3 × HA tag (Gnädig et al., 2020). Although small peptide tags of approximately 8–12 amino acids are mostly desired since they do not affect the characteristics of the target protein such as folding or protein–protein interactions (Waugh, 2005), in most studies, long peptides such as GFP or multiple repeats of the short tags have been used to improve reactivity to anti-tag antibodies. For instance, 3 × HA consisting of 27 amino acids, or spaghetti-monster tags with 10 copies of HA (Viswanathan et al., 2015), Myc (Hortua Triana et al., 2018), V5 (Rudlaff et al., 2019) or GFP (Viswanathan et al., 2015) are large peptide tags with the potential of influencing the native characteristics of target molecules. In addition, anti-tag antibodies used for detection of tagged molecules in transgenic parasites are generally less reactive in IEM than in immunofluorescence assay (IFA). As an exception, in *P. falciparum*, a double c-Myc-tagged targets could be detected by rabbit anti c-Myc polyclonal antibody in IEM, however, this antibody is no longer commercially available (Tokunaga et al., 2019). Moreover, some commercially available antibodies against the tags cross-react with parasite and host proteins, and/or have poor reactivities, while lot-to-lot variation in reactivities and specificities of polyclonal antibodies can also be an issue [reviewed in (Voskuil, 2014)]. Therefore, additional versatile protein tag options that are compatible with different molecules of interest while averting artificial effects are needed.

We recently developed the AGIA tag that contains 9 amino acids (EEAAGIARP) derived from the human dopamine receptor D1 (DRD1) (Yano et al., 2016). The AGIA tag has several advantages, namely, it is shorter than most protein tags, and free from amino acid residues susceptible to post-translational modification, and importantly, a rabbit mAb with very high affinity to the tag (*K*_d_ = 4.9 × 10^−9^ M) is established (Yano et al., 2016). In this study, we sought to investigate the applicability of the AGIA tag system to explore the ultrastructural localization of important *P. falciparum* merozoite proteins towards understanding their roles in parasite development and malaria pathogenesis.

## Materials and Methods

### *P. falciparum* Culture

*P. falciparum* 3D7 strain was a kind gift from the National Institute of Allergy and Infectious Diseases (NIAID) and cultured with type-O human erythrocytes at a hematocrit of 2% in RPMI 1640 media (Thermo Fisher Scientific, Waltham, MA, USA) and supplemented with 5% type-AB human serum, 0.25% Albumax II (Thermo Fisher Scientific), 200 mM hypoxantine (Sigma, St. Louis, MO, USA), and 10 μg/ml gentamicin (Invitrogen, Carlsbad, CA, USA) as previously described (Morita et al., 2018). The culture flask was filled with mixed gas (5% CO_2,_ 5% O_2_, and 90% N_2_).

### AGIA Tag Expression in *P. falciparum*

#### Episomal Expression of RESA-AGIA

**Figure 1:**
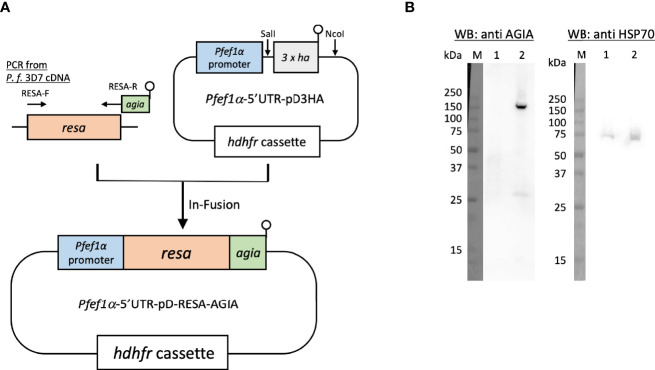
Episomal expression of RESA-AGIA in *P. falciparum* blood-stage parasites. **(A)** Schematic representation of plasmid for episomal expression of RESA-AGIA. *Resa-agia* gene was designed to be expressed under the control of *Pfef1α* promoter. *hdhfr* (human dihydrofolate reductase) gene was set for a drug selection cassette by WR 99210. **(B)** Reactivity of rabbit anti-AGIA mAb against RESA-AGIA expressing parasite. Schizont-rich parasite lysate was prepared as described (Morita et al., 2018). The schizont-rich parasite lysate was examined by Western blotting (WB) under reducing conditions probed with rabbit anti-AGIA mAb (Left). The stripped membrane was re-probed with mouse anti-HSP70 mAb as a loading control (Right). 1 × 10^7^ cells of 3D7 parasite (Lane 1) and 1 × 10^7^ cells of RESA-AGIA parasite (Lane 2) were loaded in each lane. Three independent experiments were carried out, and representative results were presented.

To examine the usage of the AGIA tag system and eventually for IEM colocalization, AGIA-tagged RESA (RESA-AGIA) was episomaly expressed in *P. falciparum*. A DNA fragment encoding full length of RESA (PF3D7_0102200: amino acids (aa) M_1_–E_1,085_) was amplified by PCR from cDNA derived from schizont-rich blood-stage *P. falciparum* 3D7 strain using primers RESA-F and RESA-R (**Table S1**). The sequence encoding AGIA tag (EEAAGIARP) was included in the RESA-R primer (underlined). *Pfef1α*-5’UTR-pD3HA (Nozawa et al., 2020) was digested by SalI and NcoI to prepare linearlized *Pfef1α*-5’UTR-pD plasmid without 3 × HA coding sequence. The amplified *resa-agia* DNA was inserted to the *Pfef1α*-5’UTR-pD by using In-Fusion HD Cloning Kit (Clontech Laboratories, Mountain View, CA, USA) to prepare *Pfef1α*-5’UTR-pD-RESA-AGIA (**Figure 1A**). For transfection with *Pfef1α*-5’UTR-pD-RESA-AGIA, the plasmid was pre-loaded into human erythrocytes in a 2 mm cuvette using a Gene Pulser Xcell Electroporation System (Bio-Rad, Hercules, CA, USA) with the conditions of 0.31 kV, 950 μF, and ∞Ω (Deitsch et al., 2001). *P.falciparum* 3D7 parasites (the parasitemia was 0.1%) were cultured with the pre-loaded erythrocytes for 4 days. After 4 days, the parasites were subsequently cultured with drug pressure of 2.5 nM WR99210.

#### AGIA-Tag Knock-In by CRISPR/Cas9

To generate AGIA-tagged multidrug resistance protein 1 (MDR1; PF3D7_0523000) endogenously expressing parasites (MDR1-AGIA), we performed CRISPR/Cas9 genome editing by co-transfection of the plasmids expressing both Cas9 and sgRNA (Cas9/sgRNA plasmid) and the linear donor DNA as previously described with some modifications (Mohring et al., 2019; Nishi et al., 2021). All the primers used are shown in **Table S1**, and the steps used are illustrated in **Figures 3A, B**.

pCas9-U6-hycen plasmid, a centromere plasmid with the Cas9 expression cassette and the sgRNA expression cassette, was generated. Specifically, cas9-expressing centromere plasmid pfcas9 (Payungwoung et al., 2018) was modified by replacing the bsd (blasticidin-S deaminase) expression cassette with hdhfr-yfcu expression cassette and adding the PfU6-driven sgRNA expression cassette using In-Fusion HD cloning kit, resulting in the pCas9-U6-hycen. Then, potential guide 20 bp RNA sequences were identified using ChopChop program (https://chopchop.cbu.uib.no) considering high efficiency and the absence of mismatched sequence to limit possibility of off-target cleavage. A pair of complementary oligonucleotides were annealed and ligated into the BsmBI-cut pCas9-U6-hycen as previously described (Nishi et al., 2021). The resultant plasmid was used for genome editing as Cas9/sgRNA plasmid. Next, a linear donor DNA was generated by overlap PCR approach as previously described (Nishi et al., 2021). The donor DNA consisted in C-terminal regions of *mdr1*, AGIA-coding sequence and 3’UTR of *mdr1*. Point mutations in the PAM (Protospacer adjacent motif) sequence and guide RNA sequence to prevent re-cleavage by the Cas9–sgRNA complex were introduced in C-terminal region of mdr1 within the donor DNA.

Cas9/sgRNA plasmid and the linear donor DNA were mixed in P3 primary cell solution (Lonza, Basel, Switzerland) and used immediately to transfect tightly synchronized schizonts using FP158 program in Nucleofector-4D (Lonza). The electroporated parasites were incubated for one day under the standard culture conditions, then pyrimethamine drug (25 ng/ml) pressure selection was applied for ten days starting one day after transfection. The MDR1-AGIA parasite clones were isolated by limiting dilution.

### Antibody Production

Rabbit antisera to the synthetic peptide derived from the C-terminal region of chloroquine resistance transporter protein (PfCRT; PF3D7_0709000 K_401_–C_419_, KKMRNEENEDSEGELTNVDC, with an additional carboxy-terminal cysteine) conjugated with keyhole limpet hemocyanin (KLH) carrier protein (Fidock et al., 2000) was commercially procured (Cosmo Bio, Tokyo, Japan). Specifically, a rabbit received a total of three immunizations with Freund Complete and Incomplete adjuvant in a 2-week interval. The antisera were collected 7 days after the last immunization.

Rabbit mAb against AGIA was prepared as previously described (Yano et al., 2016). Briefly, the antibody was expressed using the Expi293F Expression System (Thermo Fisher Scientific) according to the manufacturer’s instruction, following subcloning of anti-AGIA antibody heavy and light chains cDNAs (Takeda et al., 2015) into the pcDNA3.4 expression vector. The secreted mAb was purified from the culture medium using protein G sepharose 4 Fast Flow (GE Healthcare), and the buffer was exchanged using a PD-10 column. A mouse mAb against RESA was a kind gift from Prof. Robin F. Anders.

### Western Blotting

Purified schizont-rich parasite pellets of different clones were prepared by Percoll/sorbitol method as previously described (Arumugam et al., 2011), then lysed in Laemmli sample buffer (Bio-Rad) supplemented with 2.5% (v/v) β-mercaptoethanol. For each clone, the lysate was boiled at 95°C for 5 min, centrifuged at 10,000×*g* for 10 min at 4°C, then the supernatant was collected. The samples corresponding to 1 × 10^7^ parasites were resolved in 12.5% e-PAGEL (ATTO, Tokyo, Japan) by SDS-PAGE. The resolved proteins were electroblotted on a polyvinylidene difluoride membrane, followed by a blocking step with 5% (w/v) non-fat milk. The membranes were incubated with rabbit anti-AGIA mAb (10 μg/ml) as primary antibody. After washing, it was incubated with ECL peroxidase labeled anti-rabbit antibody (GE Healthcare) diluted at 1:10,000 for detection, and visualized with Immobilon Western Chemiluminescent HRP Substrate (Millipore, Billerica, MA) on a LAS 4000 Mini luminescent-image analyzer (GE Healthcare). The RESA-AGIA membrane was stripped with 25 mM glycine–HCl, pH 2.0, 1% (w/v) SDS and re-blocked with 5% non-fat milk, followed by incubation with mouse anti-HSP70 mAb (1:100). The parasite HSP70 signal as a loading control was detected by incubation with ECL peroxidase labeled anti-mouse antibody (GE Healthcare) diluted at 1:10,000 and captured as above. In the case of MDR1, parallel membranes were prepared; one was probed with rabbit anti-AGIA mAb as above to detect MDR1-AGIA signal, and the other was probed with rabbit anti-PfCRT antibodies (1:1,000) as a loading control and detected by ECL peroxidase labeled anti-rabbit antibody (GE Healthcare).

### Indirect Immunofluorescence Assay (IFA)

Parasites were synchronized by Percoll/sorbitol treatment and used to prepare schizont-rich blood-smears which were stored at −80°C until use. The blood-smears were fixed with 4% paraformaldehyde in PBS, permeabilized with 0.1% Triton X-100, and blocked with PBS containing 5% non-fat milk at 37°C for 30 min. The slides were then incubated with rabbit anti-AGIA mAb (10 µg/ml) and co-stained with mouse anti-AMA1 antiserum (1:100 dilution) as a microneme marker (Ito et al., 2013), mouse anti-RAP1 antiserum (1:2,000 dilution) as a rhoptry body marker (Ito et al., 2013), or mouse anti-EXP2 antiserum (1:1,000 dilution) (Morita et al., 2018) as a dense granule marker at 37°C for 1 h, followed by incubation with Alexa 488-conjugated goat anti-rabbit IgG and Alexa Fluor 568-conjugated goat anti-mouse IgG (Invitrogen) as secondary antibodies (1:1,000 dilution) at 37°C for 30 min (Morita et al., 2018). Nuclei were stained with 4’, 6-diamidino-2-phenylindole (DAPI, 4 µg/ml) (Dojindo, Kumamoto, Japan) or Hoechst 33342 (1 µg/ml) (Molecular Probes, Eugene, OR, USA). The slides were mounted in ProLong Glass Antifade reagent (Invitrogen) and observed using a confocal laser scanning microscope LSM 710 (Carl Zeiss MicroImaging, Thornwood, NY, USA).

### Immunoelectron Microscopy (IEM)

Tightly synchronized mature schizonts were isolated as previously described (Boyle et al., 2010). The parasite-infected erythrocytes were fixed with 1% paraformaldehyde/0.2% glutaraldehyde, embedded in LR White resin (Polysciences, Washington, PA, USA), and ultrathin sections were immunostained as described (Iriko et al., 2018). Specifically, rabbit anti-AGIA mAb (10 µg/ml) and mouse anti-RESA mAb (1:1,000 dilution) were used as primary antibodies. For single staining, goat anti-rabbit IgG conjugated with 15 nm Gold (BBI Solutions, Crumlin, UK) or anti-mouse IgG conjugated with 15 nm Gold (BBI Solutions) was used at a dilution of 1:40 as secondary antibodies. For double staining, goat anti-rabbit IgG conjugated with 15 nm Gold and anti-mouse IgG conjugated with 10 nm Gold (BBI Solutions) were used at a dilution of 1:40 as secondary antibodies. Samples were examined with transmission electron microscope (JEM-1230; JEOL, Tokyo, Japan).

## Results

### AGIA Tagged RESA Can Be Episomally Expressed in Transfected *P. falciparum* Parasite

Since RESA was the first, and is the most studied merozoite dense granule protein which translocates to the ring-infected erythrocyte membrane as observed by confocal laser scanning microscopy and IEM (Culvenor et al., 1991) when probed with mouse anti-RESA mAb, we sought to exploit RESA to assess the usability of the AGIA tag in parasite biology studies. Specifically, we assessed co-localization analysis by IEM using rabbit anti-AGIA mAb and mouse anti-RESA mAb in small organelles, dense granules. First, *P. falciparum* 3D7 parasites transfected with *Pfef1α*-5’UTR-pD-RESA-AGIA plasmid (**Figure 1A**), were maintained under drug pressure for constitutive expression of AGIA tagged RESA (RESA-AGIA). The expression of RESA-AGIA under the control of *Pfef1α*, a constitutive expression promoter, was assessed by Western blot probed with anti-AGIA mAb. RESA-AGIA was detected at approximately 155 kDa (**Figure 1B**, left panel lane 2). A similar signal was detected when a parallel blot was probed with anti-RESA mAb (**Supplementary Figure 1**). We also observed an additional ~25 kDa band in both blots suggesting it could be a fragment of degraded RESA. As a control, no signal of anti-AGIA mAb was detected in the non-transfected control parasite infected erythrocytes (**Figure 1B**, left panel lane 1). The membrane was further re-probed with mouse mAb against the constitutively expressed house-keeping protein, HSP70 (**Figure 1B**, right panel). The observed intensity of the HSP70 signals indicated comparable quantities of parasites were loaded in both lanes. These results indicate that the rabbit anti-AGIA mAb has negligibly low nonspecific reactivity with control 3D7 parasites.

### The RESA-AGIA Signal Overlaps With That of EXP2 in Asexual Blood-Stage Parasites

**Figure 2:**
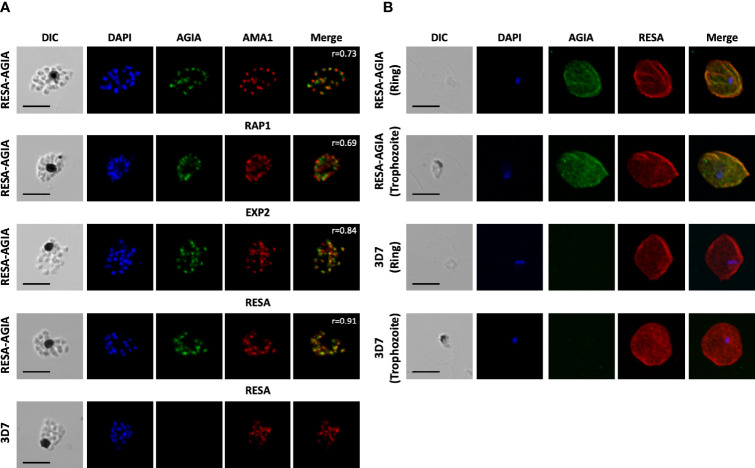
Indirect immunofluorescence assay of RESA-AGIA expressing parasites. **(A)** Blood smears for schizont stage of RESA-AGIA expressing parasites were co-stained with rabbit anti-AGIA mAb and mouse anti-AMA1 polyclonal antibodies (a microneme marker), mouse anti-RAP1 polyclonal antibodies (a rhoptry marker), mouse anti-EXP2 polyclonal antibodies (a dense granule marker), or mouse anti-RESA mAb. Pearson correlation coefficient values (r) shown in the merged panels were calculated using Zen 2010 software (Carl Zeiss MicroImaging). Scale bars indicate 5 μm. 3D7 wild-type parasite was used as a negative control. Two independent experiments were carried out, and representative results were presented. **(B)** RESA-AGIA expressing parasites at ring and trophozoite stages were co-stained with rabbit anti-AGIA mAb and mouse anti-RESA mAb. Scale bars indicate 5 μm. Three independent experiments were carried out, and representative results were presented.

To further examine the reactivity and specificity of rabbit anti-AGIA mAb against *P. falciparum* parasites expressing RESA-AGIA, an indirect immunofluorescence assay (IFA) was performed. Since the AGIA tag was fused with RESA, it was expected that RESA-AGIA would accumulate in the merozoite’s dense granules as endogenous RESA, and translocate to the ring-infected erythrocyte membrane (Culvenor et al., 1991). In schizonts stage parasites, fluorescent signals of anti-AGIA were detected as punctate pattern in each merozoite, and the signals overlapped with those of EXP2 (Pearson correlation coefficient (r) = 0.84) which is known to localize in the dense granules, and neither with anti-AMA1 (microneme; r = 0.73) nor anti-RAP1 (rhoptry; r = 0.69) (**Figure 2A**). In addition, anti-RESA mAb showed signals overlapped with anti-AGIA (r = 0.91). These results suggest that, as expected, episomally expressed RESA-AGIA was successfully translocated to the dense granules in merozoites. In the wild-type 3D7 parasites, there were no fluorescent signals of anti-AGIA (**Figure 2A**, 3D7). We further demonstrated that RESA-AGIA is also secreted to the erythrocyte cytosol and accumulated around the infected erythrocyte membrane during ring and trophozoite stage infection (**Figure 2B**) in a manner similar to endogenous RESA in the wild-type 3D7 parasites (Riglar et al., 2013). This data demonstrated that rabbit anti-AGIA mAb has specific reactivities with high signal/background ratio against human erythrocytes infected with AGIA tag-expressing 3D7 parasites.

### Endogenously Expressed Knock-In AGIA Tag is Detectable in Trophozoite Stage

**Figure 3:**
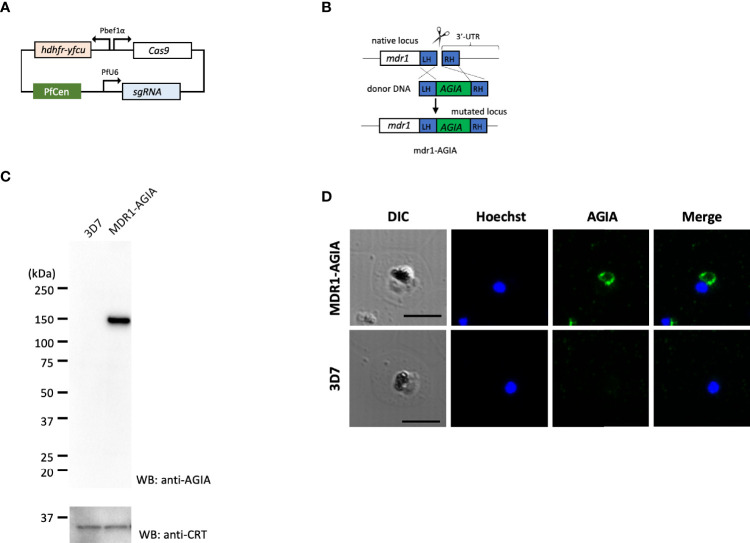
Knock-in of AGIA-tag by CRISPR/Cas9 genome editing. **(A)** Plasmid map of Cas9/sgRNA plasmid expressing both Cas9 and sgRNA. PfCen represents the centromere sequence of chromosome five of *P. falciparum*. *hdhfr-yfcu* represents the drug cassette. *hdhfr* (human dihydrofolate reductase gene) confers pyrimethamine resistance to the parasites and was used as a positive selection marker. *yfcu* is a fusion gene of yeast cytosine deaminase and uridyl-phosphoribosyltransferase. **(B)** The AGIA-tag sequence was integrated at the 3’-end of *mdr1* by homology-directed repair following CRISPR/Cas9-mediated double-strand break. **(C)** Western blotting with anti-AGIA mAb detected the expressed MDR1-AGIA protein in non-boiled sample. Anti-CRT was used as a loading control. 1 × 10^7^ cells of trophozoites were loaded in each lane. Two independent experiments were carried out, and representative results were presented. **(D)** Indirect immunofluorescence assay of MDR1-AGIA expressing parasites. Anti-AGIA mAb detected localization of MDR1-AGIA in close proximity to food vacuole of trophozoites. Scale bars indicate 5 µm. Three independent experiments were carried out, and representative results were presented.

Next, to demonstrate the usability of the AGIA tag system for the evaluation of endogenously expressed parasite proteins, we generated knock-in parasite with the AGIA tag fused to C-terminus of MDR1 (MDR1-AGIA) by CRISPR/Cas9. Generally, it is difficult to produce specific antibodies against polytopic transmembrane proteins. MDR1, an ATP-binding cassette (ABC) protein family member that localizes to the food vacuole membrane in trophozoites (Cowman et al., 1991), was therefore assessed as the representative of such proteins in malaria parasites. MDR1-AGIA parasite was generated by co-transfection of the plasmid expressing both Cas9 and sgRNA (Cas9/sgRNA plasmid) and the linear donor DNA (Mohring et al., 2019) (**Figures 3A, B**). Endogenous expression of AGIA tag in MDR1-AGIA parasites was detected by Western blotting at approximately 150 kDa (**Figure 3C**), consistent with the predicted molecular weight of MDR1 (https://plasmodb.org/plasmo/app) and in published literature (Cowman et al., 1991). PfCRT was assessed as a loading control. By IFA of trophozoite stage parasites using AGIA mAb, MDR1-AGIA was detected in close proximity of the dark mass representing hemozoin that is located in food vacuole (**Figure 3D**) as previously observed (Cowman et al., 1991). The signal/background ratio observed by Western blotting and IFA was comparable to that of RESA-AGIA analysis. These results indicate that the AGIA tag does not affect the processing and translocation of tagged proteins, RESA and MDR1.

### AGIA Tagged RESA Localizes to the Dense Granule by Immunoelectron Microscopy

**Figure 4:**
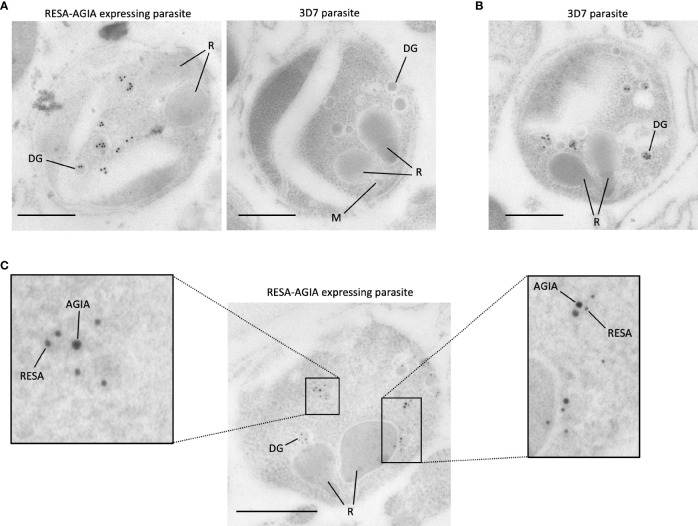
Immunoelectron microscopy of RESA-AGIA expressing parasites. **(A)** The ultrathin sections were immunolabeled with rabbit anti-AGIA mAb and goat anti-rabbit IgG conjugated with 15-nm Gold. 3D7 parasite was used as a negative control. Scale bars indicate 500 nm. R, M, and DG indicate rhoptry, microneme, and dense granule, respectively. The images are the representatives of three independent experiments. **(B)** Endogenous RESA of 3D7 parasites were immunolabeled by mouse anti-RESA mAb and goat anti-mouse IgG conjugated with 15-nm Gold. A scale bar indicates 500 nm. The image is a representative of more than three experiments. **(C)** The ultrathin section of RESA-AGIA expressing parasite was immunolabeled with rabbit anti-AGIA mAb, mouse anti-RESA mAb, goat anti-rabbit IgG conjugated with 15-nm Gold and goat anti-mouse IgG conjugated with 10-nm Gold. A scale bar indicates 500 nm. Insets show enlarged images of the part of the merozoite. The image is a representative of 18 images obtained from one experiment.

To further investigate the precise location of RESA-AGIA in merozoites, we performed IEM with mature schizonts form of RESA-AGIA parasites by staining with the rabbit anti-AGIA mAb, and goat anti-rabbit antibody conjugated with 15-nm gold particles as secondary antibody. Gold particles corresponding to the AGIA tag were detected in the dense granules of the merozoites (**Figure 4A**, left). No gold particles were observed in the merozoites of the wild-type 3D7 control parasites (**Figure 4A**, right). The IEM using wild-type 3D7 parasite stained with anti-RESA mouse mAb revealed that the gold particles were specifically localized in the dense granules (**Figure 4B**), consistent with a previous study showing endogenous RESA localization in the dense granules (Culvenor et al., 1991). Finally, to confirm the dense granules colocalization of the episomal RESA-AGIA and endogenous RESA, the RESA-AGIA parasites were stained with both anti-AGIA rabbit mAb and anti-RESA mouse mAb, and then co-labeled using anti-rabbit IgG antibodies conjugated with 15-nm gold and anti-mouse IgG conjugated with 10-nm gold. As expected, both 15-nm and 10-nm gold particles were detected in the dense granules (**Figure 4C** and inset). These results suggested that AGIA tag-fused RESA could be translocated as native endogenous RESA further confirming that the AGIA tag did not alter this process and that the tag is compatible to both confocal laser scanning microscopy and IEM localization analyses.

## Discussion

In this study, we demonstrate the application of the AGIA tag system for ultrastructural localization of RESA in the schizont merozoites. In our knowledge, this is the first report showing that the peptide tag can be detected with a monoclonal antibody in the IEM of malaria parasites. We observed that the rabbit anti-AGIA mAb could definitively detect AGIA-expressing blood-stage parasites, as illustrated by Western blotting, IFA, and IEM with high signal/background ratio. In addition, although protein tags can induce artificial effects on proteins, we show that endogenous and AGIA tagged RESA colocalized to the dense granules and then translocated to the erythrocyte membranes, indicating C-terminal fusion with the tag does not alter the localization and translocation of tagged RESA proteins in *P. falciparum*. The same holds true for food vacuole localization of MDR1 in the trophozoite stage parasites infection.

One advantage of the AGIA tag is that it is derived from human DRD1 protein expressed only in the brain (Yano et al., 2016). Thus, anti-AGIA rabbit mAb used in this study is unlikely to have cross-reactive epitopes in malaria parasite proteins. Indeed, protein BLAST analysis against malaria parasite proteins deposited in the NCBI (https://blast.ncbi.nlm.nih.gov/Blast.cgi) did not reveal any AGIA sequence match in the genome data of neither *Plasmodium* spp. parasites, nor in *Anopheles* mosquitoes. This suggests that the AGIA tag could be used to investigate the localization of other parasite proteins not only in *P. falciparum* blood stage but also in liver and mosquito stage parasites and with other parasite species. Nevertheless, we note that PfEMP1, the parasite molecules associated with the pathogenesis of the cerebral malaria, are expressed on the surface of infected erythrocyte and are ligands for erythrocytes sequestration in the brain capillary (Jensen et al., 2020). Since DRD1 is expressed mainly in the accumbens and putamen regions of the brain cell (Yano et al., 2016), in *in vivo* studies with rodent malaria AGIA tag transgenic parasites, the anti-AGIA mAb may show limited cross-reactivity in specific brain regions. Thus, although investigations are needed to confirm the usefulness of this system in such *in vivo* studies, care must be taken when interpreting the obtained findings.

Although the AGIA tag is a single epitope, multiple tags, such as 2× or 3× AGIA similar to the case of 3× HA, may be needed to improve sensitivity when assessing proteins with low expression. This may accelerate the characterization of the novel malaria parasite proteins. Taken together, the AGIA tag/anti-AGIA rabbit mAb system could be a useful tool for elucidating the subcellular localization of new and understudied proteins in malaria parasites at high resolution thus allowing in-depth evaluation of these proteins. To expand the application and adoption of this innovative technology by the wider research community, commercial availability or peer to peer sharing of the anti-AGIA rabbit mAb is under consideration.

## Data Availability Statement

The original contributions presented in the study are included in the article/**Supplementary Material**. Further inquiries can be directed to the corresponding authors.

## Author Contributions

MM and NS conceived and designed experiments. MM, NS and RK conducted experiments. MM, BK, NS and ET analyzed the data. MM, BK, NS and ET wrote the manuscript. All authors contributed to the article and approved the submitted version.

## Funding

This work was supported in part by JSPS KAKENHI (Grant number 18K15138), the Takeda Science Foundation and the Japan Agency for Medical Research and Development (AMED) under Grant numbers 21wm0325018 and 21wm0225014. The funders had no role in the study design, data collection and analysis, decision to publish, or preparation of the manuscript.

## Conflict of Interest

The authors declare that the research was conducted in the absence of any commercial or financial relationships that could be construed as a potential conflict of interest.

## Publisher’s Note

All claims expressed in this article are solely those of the authors and do not necessarily represent those of their affiliated organizations, or those of the publisher, the editors and the reviewers. Any product that may be evaluated in this article, or claim that may be made by its manufacturer, is not guaranteed or endorsed by the publisher.

## Abbreviations

mAb, monoclonal antibody; PBS, phosphate buffered saline.
