# Supplementary figures and images for: AGIA Tag System for Ultrastructural Protein Localization Analysis in Blood-Stage Plasmodium falciparum
Source: Front Cell Infect Microbiol. 2021 Dec 15;11:777291. doi: 10.3389/fcimb.2021.777291 (PMC8714843; doi:10.3389/fcimb.2021.777291)

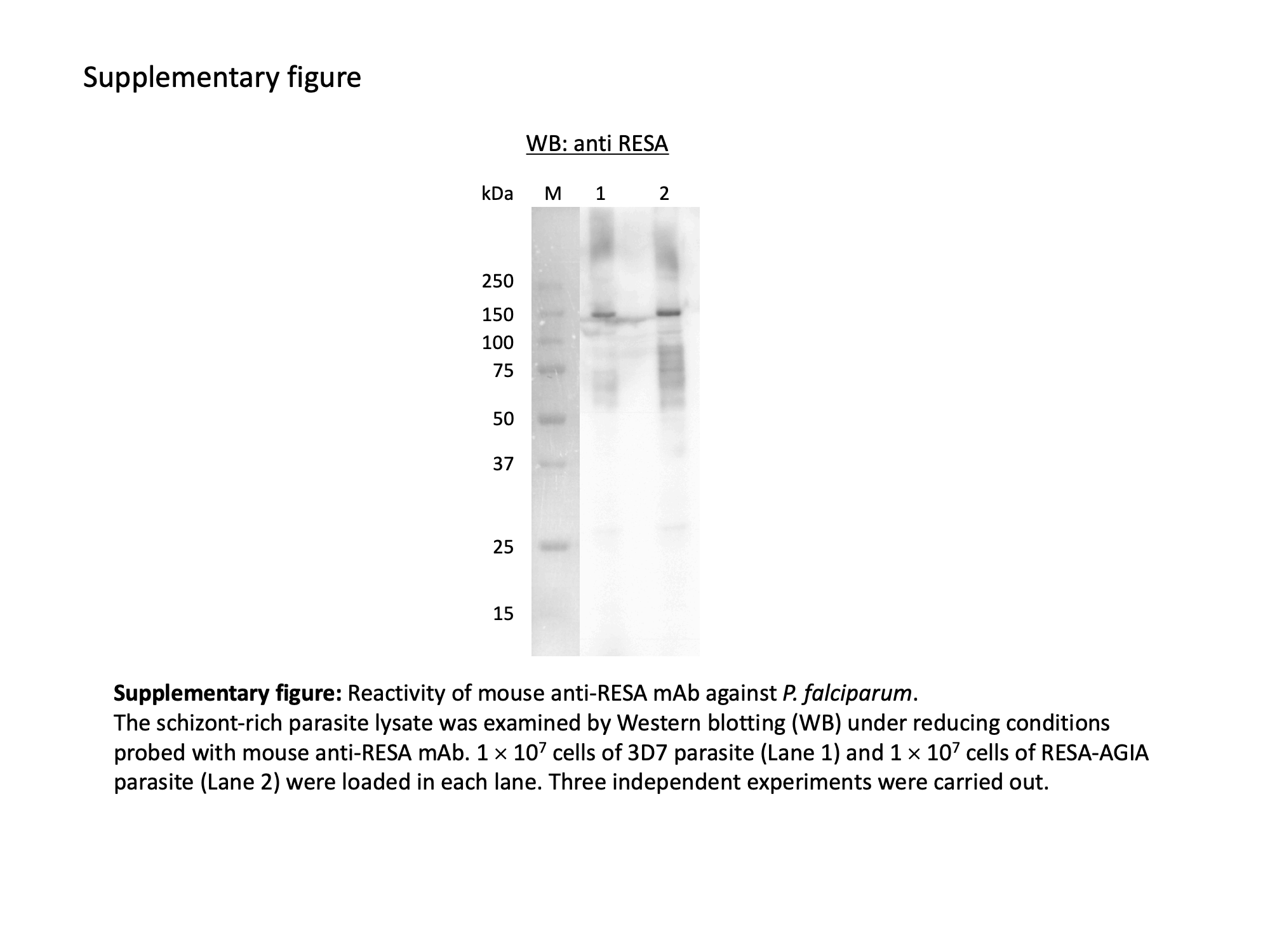

Supplement: Supplementary file 1 [file Image_1.tiff]
