# Supplementary material for: AGIA Tag System for Ultrastructural Protein Localization Analysis in Blood-Stage Plasmodium falciparum
Source: Front Cell Infect Microbiol. 2021 Dec 15;11:777291. doi: 10.3389/fcimb.2021.777291 (PMC8714843; doi:10.3389/fcimb.2021.777291)
Supplement: Supplementary file 2 [file Table_1.docx]

**AGIA tag system for ultrastructural protein localization analysis in blood-stage *Plasmodium falciparum***

Masayuki Morita^1^*, Bernard N. Kanoi^1^, Naoaki Shinzawa^2^, Rie Kubota^2^, Hiroyuki Takeda^3^ Tatsuya Sawasaki^4^, Takafumi Tsuboi^4^, Eizo Takashima^1^*

^1^Division of Malaria Research, Proteo-Science Center, Ehime University, 3 Bunkyo-cho, Matsuyama, Japan

^2^Department of Environmental Parasitology, Graduate School of Medical and Dental Sciences, Tokyo Medical and Dental University, 1-5-45 Yushima, Bunkyo-ku, Tokyo, 113-8519, Japan.

^3^Division of Proteo-Drug-Discovery, Proteo-Science Center, Ehime University, 3 Bunkyo-cho, Matsuyama, Japan

^4^Division of Cell-Free Sciences, Proteo-Science Center, Ehime University, 3 Bunkyo-cho, Matsuyama, Japan

*Corresponding authors: Division of Malaria Research, Proteo-Science Center, Ehime University, Matsuyama, Ehime 790-8577, Japan. Tel.: (+81) 89 927 9939. E-mail addresses: morita.masayuki.ls@ehime-u.ac.jp (Masayuki Morita), takashima.eizo.mz@ehime-u.ac.jp (Eizo Takashima)

**Table S1: Oligonucleotides used this RESA-AGIA and MDR1-AGIA study.**

| **Oligo name** | **Sequence** | **Description** |
| --- | --- | --- |
| RESA-F | CGGCCGCTTTGTCGAATGAGACCTTTTCATGCATATAGTTG | RESA cloning |
| RESA-R ^†^ | GGTGACGTCACCATGTTATGGTCTTGCAATTCCTGCTGCTTCTTCTTCATCATATTCTTCATTGTGTTCTTC | RESA cloning |
| mdr1-gRNA-F | TATTGTGTTCCATGTGACTGTACAA | sgRNA cloning |
| mdr1-gRNA-R | AAACTTGTACAGTCACATGGAACAC | sgRNA cloning |
| mdr1-LH-F1 | TCCGTTATATTTCAAGACCAAATGTACC | donor DNA construction |
| mdr1- LH -R1 | GTTCCATTTCGATCAGGGTTATTAAATACC | donor DNA construction |
| mdr1- LH -F2 ^#^ | ACCCTGATCGAAATGGAACgTTcGTtCAaTCACATGGAACACACG | donor DNA construction |
| mdr1- LH -R2 | CAATTCCTGCTGCTTCTTCGCTAGCTTTAGCTAATTTTACATATTTTTTATATATTCCATCTTGTGC | donor DNA construction |
| mdr1-RH-F | GAAGAAGCAGCAGGAATTGCAAGACCATGAAGCAAATCATAAATATATATATGTAATATATATATATATATGTATTTATATTTATGC | donor DNA construction |
| mdr1-RH-R | AGCAAAATATTTAGTTATTTTAAACACATTTATATTGG | donor DNA construction, Genotyping PCR |
| ef1a-F ^*^ | GCTCTTTATGCTTAAGTTTACAATTTAATATTCATAC | Plasmid construction |
| yfcu-R ^*^ | AAAAACGAACATTAAACACAGTAGTATCTGTCACCAAAG | Plasmid construction |
| dhfr3UTR-F^*^ | TTAATGTTCGTTTTTCTTATTTATATATTTATACCAATTGATTGTATTTATAACTGTAAAAATGTG | Plasmid construction |
| dhfr3UTR-R^*^ | GTTACTAGTGGGATCCCCCATCCGAAATTG | Plasmid construction |
| U6-F^*^ | CTATAGGGCGAATTGGGTACCGTAAAACGGTAAAAATAATAACACG | Plasmid construction |
| sgRNA-R^*^ | TTTTGTCGACCTCGAGAAAAAAAAGCACCGACTCGGTGCC | Plasmid construction |
| mdr1-check-F | ATTGATGTAAGAGATGATGGTGG | Genopyting PCR |
| mdr1-seq | ACAAAACTATTATTACTATTGCCCACAG | Sanger sequencing |

^†^ Underlined nucleotides represent sequence encoding AGIA tag (EEAAGIARP). ^#^ Lowercase represents introduced mutations. ^*^Underlined nucleotides represent In-Fusion tag.
